# Optimized Mortar Formulations for 3D Printing: A Rheological Study of Cementitious Pastes Incorporating Potassium-Rich Biomass Fly Ash Wastes

**DOI:** 10.3390/ma18153564

**Published:** 2025-07-30

**Authors:** Raúl Vico Lujano, Luis Pérez Villarejo, Rui Miguel Novais, Pilar Hidalgo Torrano, João Batista Rodrigues Neto, João A. Labrincha

**Affiliations:** 1 Department of Chemical, Environmental and Materials Engineering, Higher Polytechnic School of Jaén, University of Jaén, 23071 Jaén, Spain; 2 Research and Develoment Department, Cementos La Cruz, 30640 Abanilla, Spain; phidalgo@cementoslacruz.com; 3 Department of Chemical, Environmental, and Materials Engineering, Higher Polytechnic School of Linares, University of Jaén, Campus Científico-Tecnológico, 23700 Linares, Spain; lperezvi@ujaen.es; 4 Department of Materials and Ceramic Engineering, CICECO-Aveiro Institute of Materials, University of Aveiro, Campus Universitário de Santiago, 3810-193 Aveiro, Portugal; ruimnovais@ua.pt (R.M.N.); jal@ua.pt (J.A.L.); 5 Department of Mechanical Engineering, Federal University of Santa Catarina, Florianópolis 88040-900, SC, Brazil; jbrn.ufsc@gmail.com

**Keywords:** biomass fly ash, circular economy, rheology, 3D printing, cementitious material

## Abstract

The use of 3D printing holds significant promise to transform the construction industry by enabling automation and customization, although key challenges remain—particularly the control of fresh-state rheology. This study presents a novel formulation that combines potassium-rich biomass fly ash (BFAK) with an air-entraining plasticizer (APA) to optimize the rheological behavior, hydration kinetics, and structural performance of mortars tailored for extrusion-based 3D printing. The results demonstrate that BFAK enhances the yield stress and thixotropy increases, contributing to improved structural stability after extrusion. In parallel, the APA adjusts the viscosity and facilitates material flow through the nozzle. Isothermal calorimetry reveals that BFAK modifies the hydration kinetics, increasing the intensity and delaying the occurrence of the main hydration peak due to the formation of secondary sulfate phases such as Aphthitalite [(K_3_Na(SO_4_)_2_)]. This behavior leads to an extended setting time, which can be modulated by APA to ensure a controlled processing window. Flowability tests show that BFAK reduces the spread diameter, improving cohesion without causing excessive dispersion. Calibration cylinder tests confirm that the formulation with 1.5% APA and 2% BFAK achieves the maximum printable height (35 cm), reflecting superior buildability and load-bearing capacity. These findings underscore the novelty of combining BFAK and APA as a strategy to overcome current rheological limitations in digital construction. The synergistic effect between both additives provides tailored fresh-state properties and structural reliability, advancing the development of a sustainable SMC and printable cementitious materials.

## 1. Introduction

The construction industry is undergoing a fundamental transformation driven by the imperative to mitigate its significant environmental impact. Traditional building practices, characterized by high energy consumption, intensive use of raw materials, and substantial greenhouse gas emissions, are increasingly unsustainable. This situation is further exacerbated by rapid urban expansion, depletion of natural resources, and the escalating generation of construction and demolition waste, all of which underscore the urgent need for innovative and sustainable construction paradigms [1,2,3,4].

Among the emerging technologies that seek to address these challenges, additive manufacturing (AM)—commonly referred to as 3D printing—has gained recognition as a transformative approach for the construction sector. By leveraging digitally driven layer-by-layer deposition, AM enables the fabrication of complex geometries and customized structural components without the need for traditional formwork [5,6]. This not only reduces material waste and labor demand but also enhances design flexibility and accelerates the construction process. However, despite its promise, the widespread adoption of AM is currently constrained by the absence of standardized printing materials and the challenges associated with achieving controlled rheological behavior during and after the extrusion of cementitious composites [7,8,9]. Rheology describes how a material flows and deforms during mixing, transportation, and deposition and how well the parts retains their shape after printing [10]. Key factors influencing the rheological behavior of cement-based materials include temperature and humidity conditions, mix proportions, and the physical and chemical properties of the constituents, as well as processing parameters such as mixing time and energy input [11].

According to previous studies, cement pastes modified with biomass ash exhibit rheological changes that are closely linked to the chemical composition, particle size distribution, and morphology of the ash used [11]. Due to the low colloidal stability of these particles during flow, flocculation becomes a critical phenomenon influencing the paste behavior. Flocculation refers to the process by which dispersed particles aggregate into clusters through a combination of van der Waals and electrostatic forces [10]. This phenomenon significantly contributes to the thixotropic behavior of cement pastes—characterized by a reversible decrease in viscosity under constant shear, followed by structural rebuilding once the shear stress is removed. Flocculation is also considered one of the main mechanisms behind shear-thinning behavior, where viscosity decreases under high strain rates (between 100 and 10^2^ s^−1^), a condition commonly encountered in mixing and flow operations in fresh-concrete applications [12].

**Figure 1:**
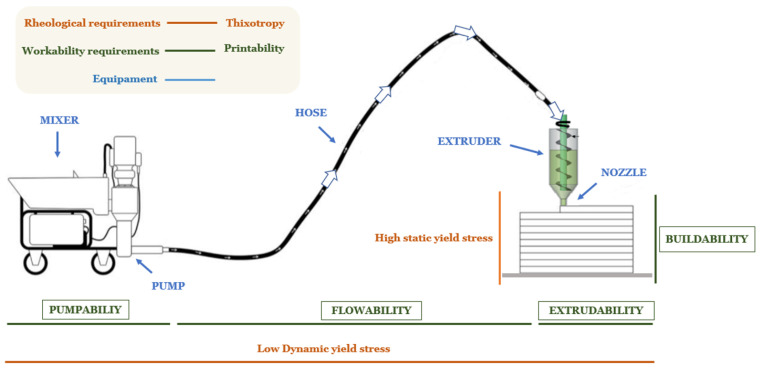
Integrated analysis of rheological and workability requirements for enhanced 3D printing of cementitious materials.

Extrusion-based 3D printing, also known as material extrusion (ME), is currently the most mature and widely adopted technique for additive construction. This method requires the development of highly specialized mortar formulations that meet stringent requirements in the fresh state. These formulations must achieve an optimal balance between flowability—necessary for pumping and extrusion—and buildability, which ensures shape retention and structural integrity post-deposition. The critical rheological parameters governing these behaviors include yield stress, which dictates the ability to support successive layers; plastic viscosity, which controls ease of flow through the nozzle; and thixotropy, which facilitates rapid structural rebuilding after shear cessation [13,14,15,16]. All these parameters within the printing process are illustrated in Figure 1. Additionally, the study considers the phenomenon of pseudothixotropy in cementitious systems, acknowledging that the time-dependent rheological behavior results not only from reversible flocculation and deflocculation of particles but also from the irreversible effects of hydration reactions. This dual contribution is particularly relevant for printable mortars, where structural rebuilding and setting occur simultaneously.

In parallel, the construction industry is actively pursuing the integration of supplementary cementitious materials (SCMs) derived from industrial residues to enhance the sustainability of cement-based systems. Biomass fly ash (BFA), a by-product generated during the combustion of organic materials in biomass power plants, is gaining traction as a viable SCM. Its adoption contributes to reducing the clinker factor, promotes waste valorization, and, in many cases, imparts pozzolanic activity to the binder matrix [17,18,19]. Nevertheless, BFAs exhibit a high degree of variability in terms of chemical and mineralogical composition, particle size distribution, and amorphous content, which critically affect their reactivity and compatibility with cementitious systems [20].

Despite an expanding body of literature on the use of generic BFA in conventional cement and concrete applications, the specific behavior of potassium-rich biomass fly ash (BFAK) remains largely unexplored. BFAK originates predominantly from the combustion of agricultural residues, such as olive pomace and almond shells—residues abundantly available in countries like Spain, where over 1.4 million tonnes of biomass ash are produced annually [21]. Unlike more commonly studied BFAs, BFAK is characterized by high concentrations of soluble alkali salts, particularly potassium-based compounds (e.g., KCl, K_2_CO_3_), which exert unique effects on cement hydration mechanisms and fresh-state rheological behavior. These characteristics differentiate BFAK substantially from conventional pozzolanic ashes and complicate its direct incorporation into standard cementitious matrices.

The potential incorporation of BFAK into mortars designed for 3D printing introduces both technical challenges and opportunities for performance enhancement. On one hand, the presence of reactive alkali phases may induce delayed hydration, false setting phenomena, or instability in the extrusion process. On the other hand, these same features may be harnessed to modulate hydration kinetics, enhance buildability through rapid structural recovery, and improve thixotropic response. Importantly, no prior studies to date have examined the integration of BFAK into 3D-printable mortars or explored its interaction with admixtures designed to regulate rheological performance [22,23].

However, limited studies have systematically evaluated the interaction between thixotropy, yield stress, and buildability in the context of sustainable cementitious formulations for 3D-printing applications. This work aims to address this gap by investigating the effects of BFAK incorporation into extrusion-based 3D-printing formulations, both independently and in combination with an air-entraining plasticizer (APA). The novelty of the work lies in the comprehensive evaluation of how BFAK modifies key fresh-state and early-age properties—namely, rheology, hydration kinetics, and buildability—and in the demonstration of how these effects can be systematically controlled through the use of APA. The findings contribute to the development of tailored, sustainable cementitious composites for additive manufacturing and propose a viable valorization pathway for high-potassium biomass ash wastes, currently underutilized in the construction sector.

## 2. Materials and Methods

### 2.1. Raw Materials

#### 2.1.1. Description of Raw Materials

The primary binder utilized in this research is white Portland cement (WPC), provided by Cementos La Cruz (Murcia, Spain), in accordance with the UNE-EN 197-1 [24] specification. This cement is distinguished by its high early mechanical strength and reduced concentrations of iron and manganese oxides, which is responsible for its whiteness. Mineralogically, the cement is predominantly composed of alite (C3S) and belite (C2S), accompanied by minor quantities of gypsum, added to regulate the setting kinetics.

As a supplementary cementitious material, a biomass fly ash rich in potassium (BFAK) is incorporated. This by-product was sourced from the ENCE La Loma biomass power facility in Villanueva del Arzobispo (Jaén, Spain), where burned olive stones used as biomass fuel are thermally processed.

An air-entraining plasticizing admixture (APA), also supplied by Cementos La Cruz (Murcia, Spain), was introduced in varying dosages to improve the fresh-state rheological behavior and optimize the balance between fluidity and structural retention. The effect of the APA was analyzed both in isolation and in conjunction with BFAK to elucidate synergistic mechanisms.

As the fine-aggregate component, a limestone sand (LS) with a maximum particle size below 2 mm was employed. This material, provided by Cementos La Cruz (Murcia, Spain), offers high purity and consistent granulometry.

#### 2.1.2. Physical and Chemical Characterization of Raw Materials

**Figure 2:**
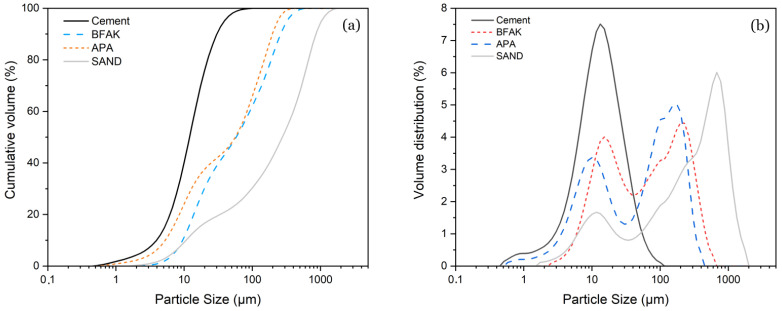
Particle size of raw materials: (**a**) Cumulative volume (%) and (**b**) volume distribution (%).

**Figure 3:**
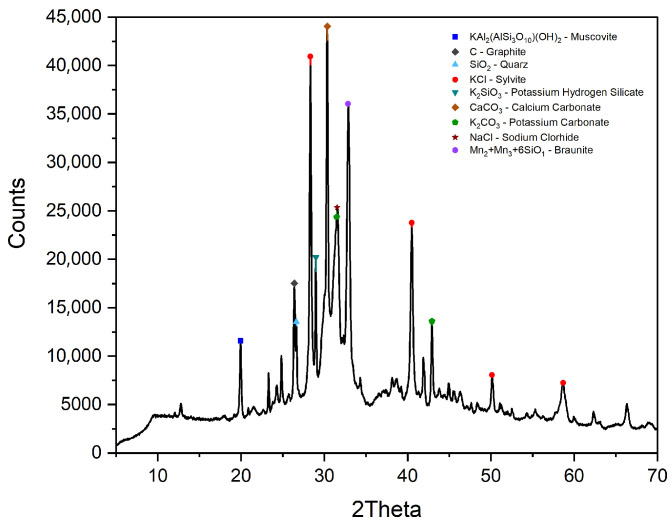
X-ray diffraction of BFAK.

To ensure the compatibility and performance of the selected materials in cementitious matrices, a comprehensive suite of physicochemical analyses was conducted:

- X-ray fluorescence (XRF): The oxide compositions of WPC and BFAK were quantified using XRF. Raw materials were subjected to an initial conditioning process to ensure uniformity, which was confirmed prior to characterization. Table 1 summarizes the major oxides, accompanied by standard deviations to reflect the reproducibility of the measurements.
- Particle size distribution (PSD): Laser diffraction analyses were conducted using a Malvern Zetasizer Nano ZS under dry dispersion conditions. The cumulative volume curve (Figure 2a) shows that WPC exhibits a narrow, fine distribution (1–30 µm). In contrast, BFAK and APA display broader ranges, with bimodal distributions evident in the volume fraction curve (Figure 2b), characterized by fine particles (10 µm) and larger fractions (50–100 µm). The sand curve is also bimodal, with the dominant peak between 900 and 1000 µm.
- Scanning electron microscopy (SEM): The microstructure of the biomass fly ash was studied using a JEAL SM 840 model (Akishima, Tokyo, Japan) assisted by energy dispersive X-ray spectroscopy (EDS). The sample was carbon-coated using a JEOL JFC 1100 sputter coating.
- X-ray diffraction (XRD): The mineralogical characterization of the samples was performed using X-ray diffraction (XRD), a Bruker D2 PHASER diffractometer (Bruker, Billerica, MA, USA), operating with Cu Kα radiation (λ = 1.5406 Å) in θ–2θ configuration. The scan was conducted over a 2θ range of 5° to 70°, with a step size of 0.02° and a counting time of 2 s per step; the corresponding diffractogram is presented in Figure 3. Rietveld refinement was used; BFAK consists of approximately 64% amorphous phase. The crystalline phases identified included sylvite (KCl) and potassium carbonate (K_2_CO_3_). Quartz (SiO_2_), muscovite (KAl_2_(AlSi_3_O_10_)(OH)_2_), and calcium carbonate (CaCO_3_) were also detected.

### 2.2. Conceptual Research Method

**Figure 4:**
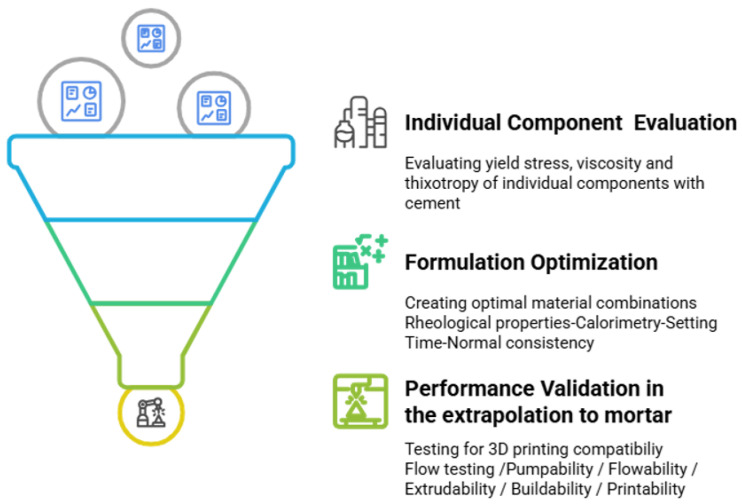
Schematic workflow for rheological characterization and optimization of biomass fly ash on cementitious materials for additive manufacturing.

The methodology used in this study follows a systematic and sequential framework designed to evaluate and optimize the rheological behavior of cementitious materials for 3D-printing applications, as shown in Figure 4. Each stage builds upon the outcomes of the previous one, ensuring a coherent and progressive approach to material development.

The process begins with the analysis of the rheological properties of the individual components (Figure 4), including cement combined with additives (APA) and biomass fly ash (BFAK). Critical parameters such as yield stress, viscosity, and thixotropy are evaluated to understand the specific contributions of each component to the overall behavior of the material. This initial stage isolates the effects of each component.

The results of the initial stage guide the formulation of optimal material combinations. In this stage (Figure 4), the rheological properties of the complete formulations are reassessed. Complementary tests are performed including determinations of normal/suitable consistency, setting time, and calorimetric behavior.

The final stage involves the optimized formulations and aims to validate their performance in large-scale 3D printing. Key parameters such as flow testing, pumpability, flowability, extrudability, buildability, and printability are evaluated. These tests ensure that the material can maintain consistent flow, deposit precise layers, and provide structural stability to support subsequent layers. The results of this stage confirm the feasibility and reliability of the formulations for industrial use.

### 2.3. Paste Characterization

#### 2.3.1. Paste Mix Formulations

The Table 2 shows the composition of the cement paste samples used for the rheological tests. Each sample maintains a fixed water-to-cement ratio (w/c = 0.4). Prior to finalizing the composition of the optimal (OPT) formulations, preliminary rheological tests were conducted on a set of mixtures containing four different dosages of each individual component BFAK and APA. These dosage intervals were selected to cover a representative range of influence on fresh-state behavior. From these preliminary trials, the two intermediate proportions for each material were chosen for further combined testing. This sequential optimization strategy ensured that the selected formulations were not arbitrary but derived from a systematic evaluation of rheological criteria relevant to 3D-printing applications.

#### 2.3.2. Rheological Performance and Relevance for 3D Printing

**Figure 5:**
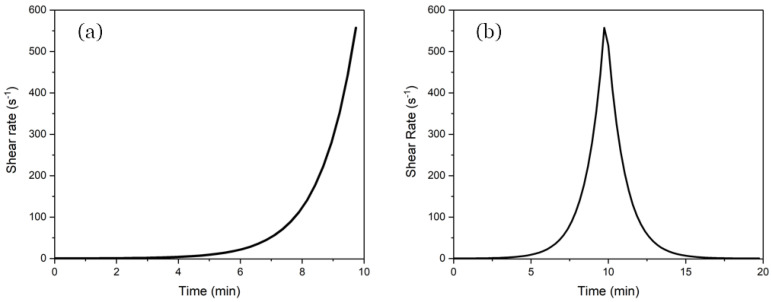
(**a**) Logarithmic shear-rate ramp; (**b**) logarithmic up and down thixotropy model.

A Kinexus lab+ rheometer was employed to assess parameters such as yield stress, viscosity, and thixotropic behavior. The paste samples were prepared by mixing with distilled water (w/c = 0.4) for 3 min at 800 rpm, ensuring a consistent hydration process. Rheological tests were performed using two complementary methods to characterize yield stress and thixotropy. The tests were carried out under controlled conditions at a consistent temperature of 25 °C and a relative humidity between 60 and 70%. Yield stress was analyzed by performing a shear-rate ramp test, where the shear rate progressively increased from 0.1 s^−1^ to 600 s^−1^ over a period of 10 min, as shown in Figure 5a. This test was repeated at 0, 20, and 40 min after mixing, with time zero defined as 7 min after initial contact between the cement and water (w/b 0.4) to ensure uniform hydration initiation in all tests.

The Herschel–Bulkley model [25] was applied to fit the results of the shear-rate ramp test and to estimate the yield stress and viscosity values, using Equation (1).

(1) $$\tau =\tau_{0}+K{\dot{\gamma }}^{n}$$

where τ is the shear stress (Pa), $\tau_{0}$ is the yield stress, *k* is the consistency index, γ is the shear rate (s^−1^), and *n* is the flow behavior index.

To estimate thixotropy, an “up and down” cycle was applied. In this test, the shear rate was initially increased from 0.1 $s^{-1}$ to 600 $s^{-1}$ over 10 min, followed by a reverse ramp from 600 s^−1^ to 0.1 s^−1^ over the next 10 min (Figure 5b). This cycle captures the structural breakdown and rebuilding behavior of the material under both increasing and decreasing shear, allowing us to quantify thixotropic recovery and giving information about the stability of the material upon extrusion [12].

#### 2.3.3. Setting Time

The setting times of the cementitious pastes were determined following the UNE-EN 196-3:2017 [26] standard, which establishes a precise method for measuring the initial and final setting times using the Vicat needle apparatus. According to this standard, the initial setting time is recorded when the paste reaches sufficient resistance to prevent full needle penetration, marking the onset of stiffening. The final setting time is observed when the paste reaches a point where the needle no longer leaves a visible impression, indicating structural solidification. The difference between the initial and final setting times corresponds to the open time, the period where the material is workable.

#### 2.3.4. Isothermal Calorimetry

The Calmetrix I-Cal ultra isothermal calorimeter was used to perform calorimetric analysis, using two different approaches to evaluate the effect of biomass fly ash on the cement hydration energy and kinetics during the first two days [27]. In the first approach, all mixtures were prepared with a fixed water-to-cement ratio of 0.4 to isolate and observe the impact of biomass ash on hydration compared to the reference cement sample. In the second approach, optimized formulations were prepared showing the normal/consistency for printing, adjusted with variable amounts of water. This method provided insight into the hydration heat and thermal behavior of the material in a practical application scenario, reflecting the actual conditions under which the optimized mixes would be used.

### 2.4. Mortar Characterization

#### 2.4.1. Mortar Mix Formulations

The formulations tested at this stage were developed by extrapolating the findings acquired from the previous stage (using pastes). The sand-to-cement ratio was fixed at 0.6 (LS/WPC). Table 3 details the compositions, using the same designations of the pastes.

#### 2.4.2. Flow Testing

To evaluate the consistency, spreadability, and dispersion characteristics of the mortar formulations, a standard flow table test was used. These properties ensure that the material flows uniformly during extrusion without segregation. The samples were subjected to 15 standardized jolts on the flow table and their spread diameters were recorded. Based on these results, the water content in the mortar was adjusted to achieve optimal rheological behavior (the named normal/suitable consistency), ensuring that the material presented suitable workability for extrusion and layering in 3D-printing applications.

#### 2.4.3. Calibration Cylinder Fabrication

**Figure 6:**
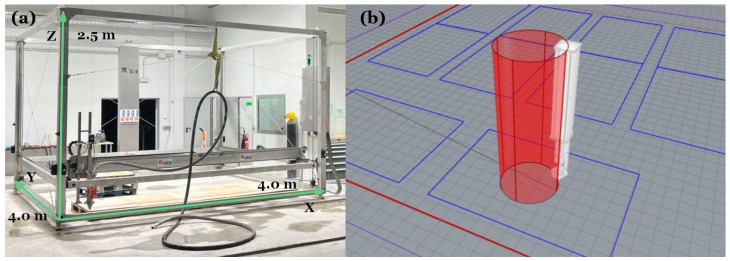
Experimental setup for 3D printing of calibration cylinders: (**a**) Large-scale 3D printer at Cementos La Cruz, and (**b**) digital model of the calibration cylinder.

To assess material performance during additive manufacturing, a calibration cylinder was fabricated using a 3D LAB printer located at Cementos la Cruz in Abanilla, Murcia (Figure 6a). The test aimed to evaluate critical characteristics, including extrudability, pumpability, flowability, buildability, and printability. These parameters have been identified in previous studies as crucial in defining the suitability of cementitious materials for additive manufacturing [28].

The cylinder was designed with a base diameter of 440 mm. These dimensions were defined using a digital model (Figure 6b), which provided a precise representation of the intended geometry. The model guided the path of the printer, ensuring consistent layer deposition and enabling an accurate comparison between the digital design and the physical structure.

During fabrication, the printer operated at a constant speed of 100 mm/s, extruding the mortar through a nozzle with a diameter of 20 mm and a layer height of 8 mm. A pump system continuously supplied the mortar to maintain uniform flow. Observations during the printing process focused on the ability of the material to maintain its geometric integrity, support successive layers, and produce a structurally sound and dimensionally accurate cylinder. To assess the repeatability and variability of the printing behavior, three calibration cylinders were printed for each formulation under identical conditions.

## 3. Results and Discussion

### 3.1. Rheology Behavior of Individual and Combined Additives in Cement Paste: Yield Stress and Consistency

#### 3.1.1. Yield Stress and Consistency

**Figure 7:**
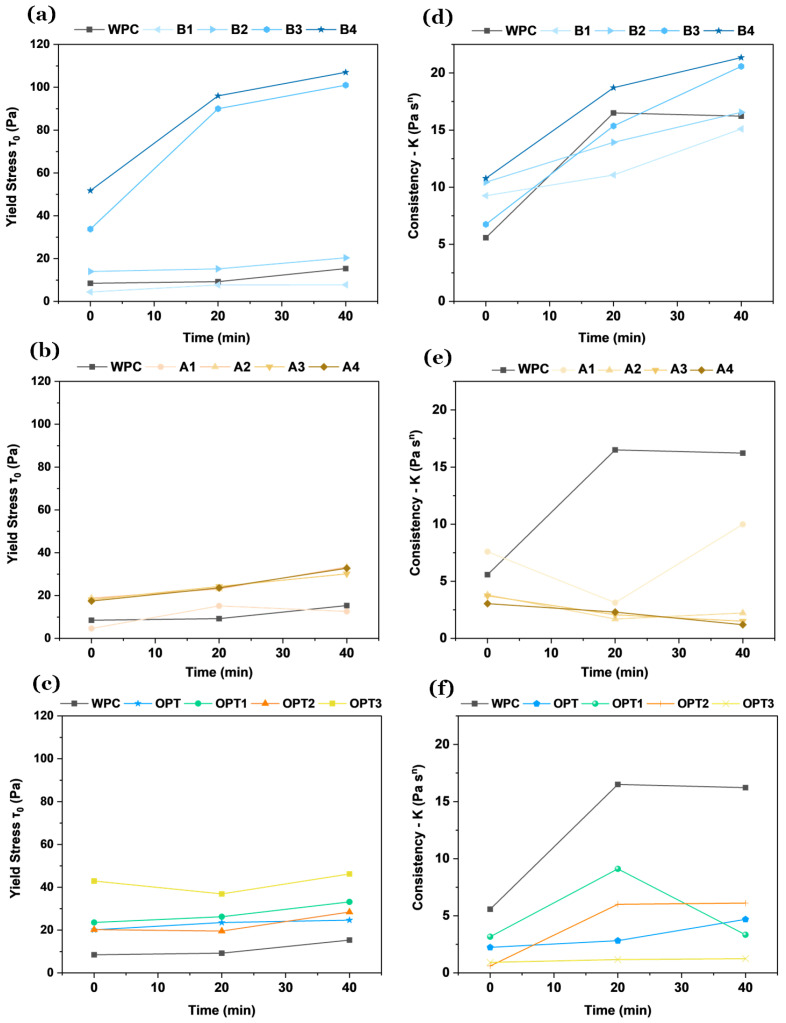
Time-dependent evolution of yield stress ($\tau_{0}$) and consistency (K) for cementitious mixtures: (**a**) APA yield stress; (**b**) BFAK yield stress; (**c**) combined APA and BFAK yield stress; (**d**) APA consistency; (**e**) BFAK consistency; and (**f**) combined APA and BFAK consistency.

The results presented in Figure 7 demonstrate the evolution of yield stress ($\tau_{0}$) and consistency (K) over time for cementitious mixtures containing APA, BFAK, and their combinations. These variables highlight the distinct and complementary effects of additives.

The results of the yield stress (Figure 7b) show the distinct effect of APA, where higher doses (A3 and A4) produce a significant increase over time. This behavior reflects the ability of APA to enhance structural recovery through its air-entraining effect, which promotes the formation of micro bubbles that strengthen the internal structure of the material. The effect of such additives in preventing the occurrence of segregation is well known [29]. However, BFAK also contributes to an increase in yield stress, as shown in Figure 7a. The incorporation of potassium-rich biomass fly ash (BFAK) into cementitious mortars significantly influences their rheological properties, particularly yield stress and viscosity, which directly affect workability and buildability in additive manufacturing applications. The effect of BFAK addition leads to a pronounced increase in consistency, expressed as yield stress, for the same amount of additive incorporated into mortars. Compared to air-entraining plasticizer (APA), BFAK exhibits a distinct behavior at higher dosages, particularly at early ages. While both additives show a gradual increase in yield stress for dosages up to 1%, BFAK additions above this threshold result in a rapid and substantial increase within the first minutes, reaching approximately 110 Pa for a 4% addition. This increase is particularly noticeable after 20 min, with a more pronounced stiffening effect in mortars containing BFAK than in those with the APA.

The increase in yield stress and viscosity observed with BFAK is attributed to its chemical composition, particularly to the relatively high concentrations of potassium-bearing compounds, such as sylvite (KCl) and potassium carbonate (K_2_CO_3_). When in contact with water and cement, these compounds react to form alkaline sulfates, as shown in Equations (2) and (3), and these lead to the development of complementary hydration phases such as Aphthitalite (K_3_Na(SO_4_)_2_), as expressed in Equation (4). The formation of these additional phases improves the cohesion of the cementitious matrix, leading to an accelerated stiffening effect, similar to the false setting [30]. This behavior improves the material’s ability to retain its shape post-extrusion. In addition to its influence on extrusion, the yield stress also governs the ability of the printed layer to support subsequent layers without deformation. A sufficiently high yield stress ensures that each layer maintains its geometry under the weight of the upper layers, directly contributing to buildability and the overall stability of the structure during printing.

(2) $${\mathrm{KCl}}_{\left(s\right)}\overset{\;H_{2}O\;}{\to }K_{\left(aq\right)}^{+}+{\mathrm{Cl}}_{\left(aq\right)}^{-}$$

(3) $$K_{2}{\mathrm{CO}}_{3_{\left(s\right)}}\overset{\;H_{2}O\;}{\to }2K_{\left(\mathrm{aq}\right)}^{+}+C{O_{3}^{2-}}_{\left(\mathrm{aq}\right)}$$

(4) $$3K_{\left(\mathrm{aq}\right)}^{+}+{\mathrm{Na}}_{\left(\mathrm{aq}\right)}^{+}+2{{\mathrm{SO}}_{4}^{2-}}_{\left(\mathrm{aq}\right)}\to K_{3}\mathrm{Na}{\left({\mathrm{SO}}_{4}\right)}_{2_{\left(s\right)}}$$

where the alkaline sulfates $K^{+}$ come mainly from KCl and K_2_CO_3_, ${\mathrm{Na}}^{+}$ from cement and BFAK, and ${\mathrm{SO}}_{4}^{2-}$ from gypsum.

Research on alkali-activated materials has shown that potassium-rich fly ashes play a key role in regulating early-age hydration kinetics [31,32]. The high solubility of potassium salts promotes rapid ion exchange and nucleation of secondary hydration phases, reinforcing the paste’s structural framework. Consequently, pastes with higher BFAK content exhibit a progressive increase in yield stress over time, confirming the strong rheological influence of potassium-based additives in cementitious materials. The effects observed herein align with previous studies on alkali-activated binders, where potassium-enhanced systems have demonstrated superior structural integrity and long-term stability [31,32].

**Figure 8:**
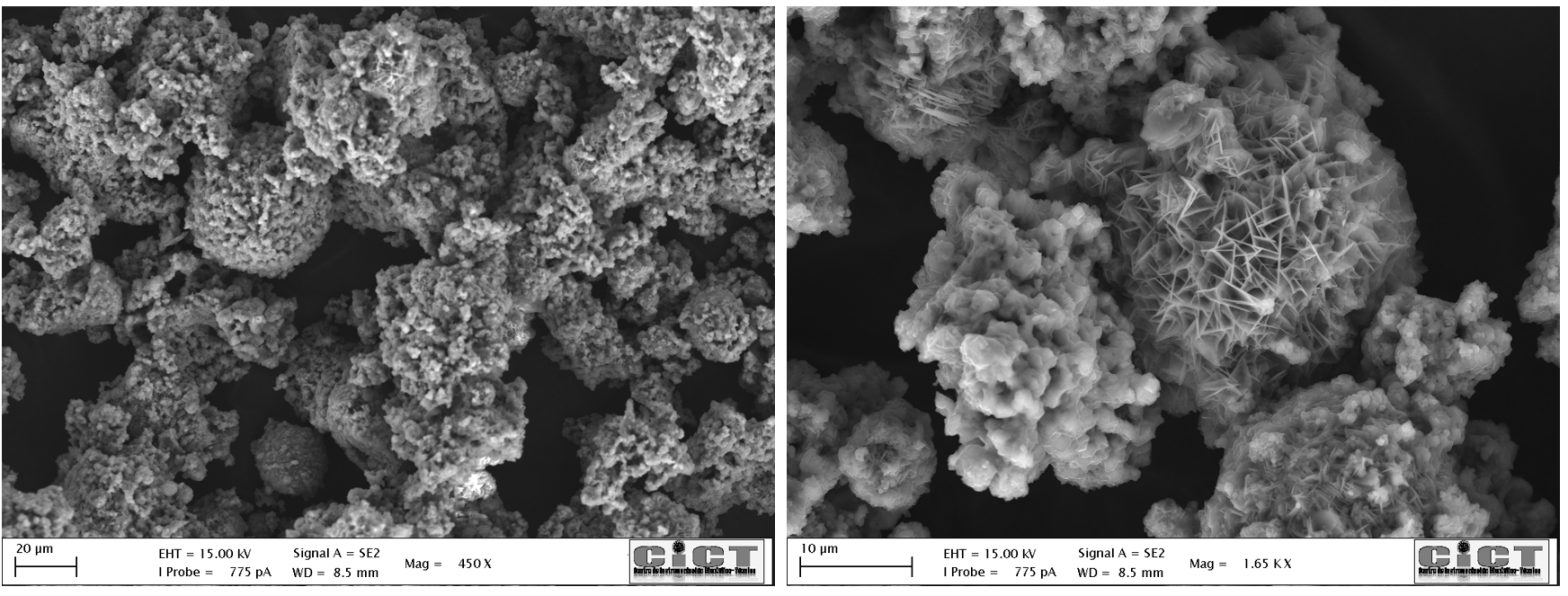
SEM of BFAK particles at different magnifications.

The consistency results in Figure 7d confirm the strong influence of BFAK on the viscosity of the mixtures. Higher doses of BFAK (B3 and B4, Figure 7d) promote a notable consistency increase over time. In addition to the mentioned chemical effects, the role of BFAK is also affected by physical contributions. In fact, particles exhibit non-spherical shapes with angular edges and a highly irregular surface profile (Figure 8), so the total surface area of the irregular interfaces also rises, reducing their sliding ability. The increase in interparticle friction leads to more energy being required for flow. So, in general, effects are predictably more pronounced when the BFAK amount increases.

In contrast, the addition of APA generally leads to a reduction in the consistency of the formulations. Due to its surfactant nature, the additive also acts, to some extent, as a deflocculant (i.e., an additive that reduces the viscosity/consistency of dispersions). Furthermore, APA appears to inhibit hydration reactions to some degree, as the consistency does not change over time, unlike the formulation without the additive. Lower APA dosages (A1 and A2) show an impact on consistency, while higher dosages (A3 and A4) promote stabilization. At low APA concentrations, given the low presence of hydration products at early ages, it can be stated that the rheological properties are mainly influenced by physical forces between particles. The combined formulations show a balanced consistency trend, where neither additive completely dominates, but BFAK plays a more prominent role in maintaining viscosity.

In formulations with both additives (Figure 7c,f), their roles seem complementary. APA reduces the viscosity of the mixture compared to the reference (WPC) because of its plasticizing action. At the same time, it increases the yield stress, enhancing structural recovery and stability. BFAK promotes cohesion and increases viscosity, with a steady growth in the yield stress and consistency as its dosage increases. Among the formulations prepared with both additives, OPT3 exhibits the most pronounced increase in the yield stress, reflecting the synergistic effect of APA and BFAK. This formulation also shows a noticeable decrease in consistency compared to other combinations, balancing flow resistance and structural build-up.

#### 3.1.2. Pseudothixotropy and Structural Buildability

The rheological behavior discussed in this section reflects the pseudothixotropic nature of cementitious pastes, where both reversible and irreversible phenomena contribute to the structural evolution under shear. The reversible component is associated with flocculation–deflocculation cycles governed by particle interactions, whereas the irreversible contribution arises from hydration reactions that progressively alter the internal structure.

**Figure 9:**
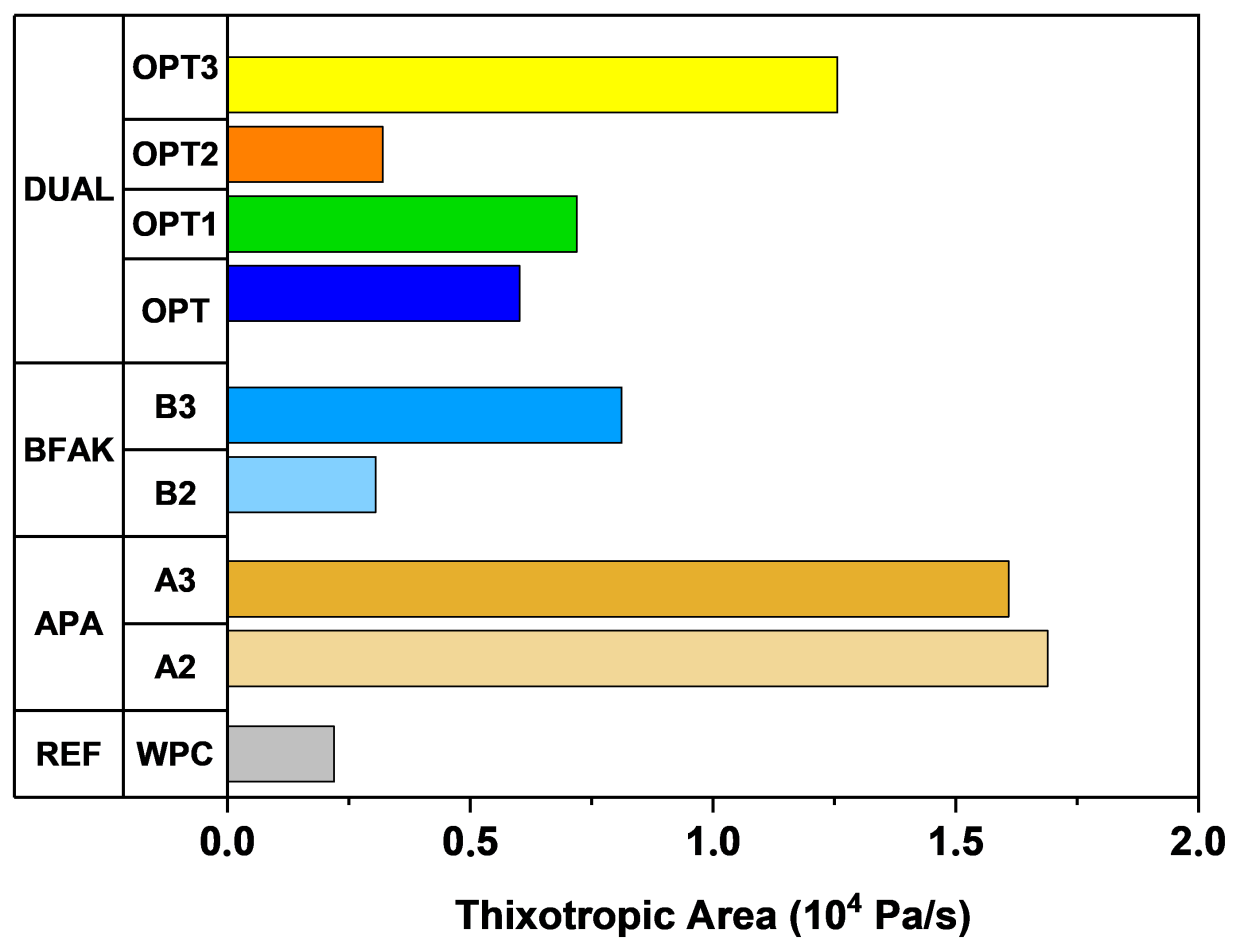
Thixotropy area of individual and dual combinations.

Figure 9 illustrates the thixotropic area between 50 and 500 s^−1^ for the mixtures evaluated, highlighting the structural recovery capabilities of formulations containing APA, BFAK, and their combinations. The results demonstrate the distinct contributions of each additive to the thixotropic behavior, as well as the synergistic effect observed in mixtures combining the two.

APA plays a dominant role in enhancing thixotropy. Its dispersing and air-entraining action improves particle distribution and stabilizes flocculated structures within the paste, allowing rapid reformation of the microstructure once shear is removed. This effect is reflected in a consistent increase in the thixotropic area with higher APA content. BFAK also contributes to thixotropic recovery by increasing internal cohesion through its physicochemical interaction with the cement matrix. Although its individual effect is less pronounced than that of APA, its contribution becomes more significant at higher dosages.

The combination of APA and BFAK results in a synergistic improvement, as observed in formulations that exhibited the highest thixotropic areas. The thixotropic behavior of each formulation plays a critical role in the success of 3D printing applications. Specifically, a greater thixotropic area indicates a higher structural recovery after shear, which enhances the material’s ability to retain its shape once deposited. This property contributes to the stacking stability of successive layers, preventing lateral deformation or collapse between print passes. In this study, the formulations that exhibited wider thixotropic areas showed improved vertical alignment and shape fidelity during printing, as reflected in the results shown in Section 3.3.

### 3.2. Setting Time and Hydration Kinetics of Cementitious System

**Figure 10:**
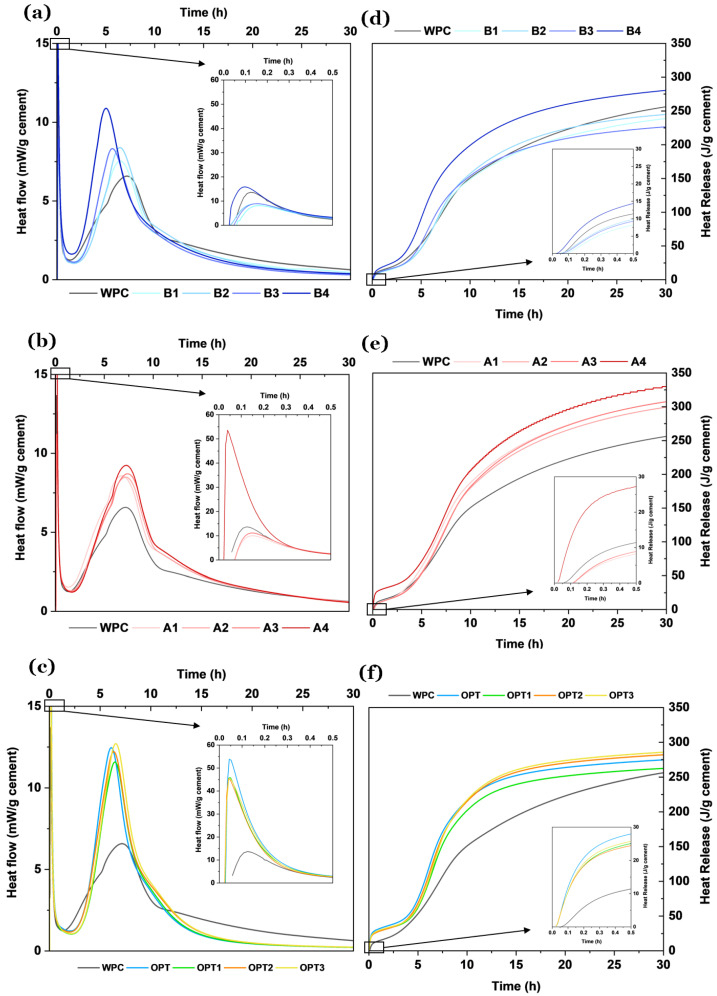
Hydration kinetics and heat release profiles of cementitious systems: (**a**,**d**) BFAK-modified pastes, (**b**,**e**) APA-modified pastes, and (**c**,**f**) combined BFAK and APA formulations.

Isothermal calorimetry of the cement pastes incorporating potassium-rich biomass fly ash waste (BFAK) (Figure 10a,d) reveals distinct hydration kinetics compared to ordinary Portland cement (WPC). The thermograms exhibit the canonical four-stage hydration profile, in agreement with the established literature [33].

The BFAK-containing formulations exhibit an advancement in the onset of the acceleration phase, with the primary hydration peak occurring earlier than in the WPC reference. The initial exothermic signal—detected within the first 0 to 0.1 h and emphasized in the zoomed-in portion of Figure 10a—corresponds to the rapid dissolution of reactive phases, specifically alite (C3S) and tricalcium aluminate (C3A), along with the formation of ettringite through reactions between C3A and sulfate species in the pore solution [34,35].

As the BFAK dosage increases—up to 4 wt.%—this early hydration peak becomes more pronounced, and its position shifts slightly toward earlier times, indicative of enhanced reactivity. The subsequent induction period (0.1–1.25 h) is characterized by a sharp reduction in heat evolution across all formulations, though its duration shows a clear dependence on BFAK content. In low-to-moderate BFAK dosages (B1–B3), the induction and subsequent acceleration periods are slightly prolonged relative to WPC, and the primary peak is shifted to earlier times. This behavior is ascribed to the transient formation of a passivating layer on cement grain surfaces, likely stabilized by potassium ions, which momentarily delay active hydration onset. In contrast, at higher BFAK content (B4), the induction phase shortens significantly and leads into a more intense and earlier main heat flow peak. This is attributed to the elevated presence of K_2_O, which enhances ionic mobility, facilitates silicate dissolution, and thereby accelerates the hydration process. Such behavior mirrors that observed in alkali-activated systems with high-alkali SCMs, where hydration is rapidly initiated due to increased pH and ionic conductivity.

Additionally, the presence of sylvite (KCl) alters the ionic composition of the pore solution by reducing the amount of freely available water, which influences the early-age rheological properties of the system. The elevated heat evolution in BFAK-rich systems further confirms the high reactivity of these ashes, consistent with previous findings that biomass ashes increase the heat released per gram of cement during hydration [36,37].

In contrast to the findings presented in this study, previous investigations into biomass ashes with high calcium oxide (CaO) contents have reported delayed hydration behaviors. Skevi [34] documented a significant shift in the exothermic peak of the acceleration period toward later times when high-CaO ashes were incorporated into cement. Similarly, Fort et al. [38] observed that biomass ashes rich in SiO_2_, CaO, and Al_2_O_3_, but with low potassium content, led to reduced total heat release and delayed hydration kinetics—particularly under conditions where higher substitution levels of cement by ash were employed.

Ultimately, the presence of potassium-bearing mineralogical species appears to be the dominant factor accelerating the hydration process during the acceleration stage. Following the main exothermic peak, a gradual decline in heat release was observed for all samples, a trend partially attributable to the retarding influence of fly ash on early hydration. While it might be hypothesized that other mechanisms—such as pozzolanic activity due to filler effects, or the hydration of free lime (periclase) and CaO/Al_2_O_3_-rich phases could contribute to increased heat evolution, these can be confidently excluded in this case. The relatively large particle size of BFAK reduces the likelihood of significant nucleation effects, and the content of free lime or reactive CaO and Al_2_O_3_ in the ash is not substantial.

Therefore, the increased heat flow and cumulative heat release observed in BFAK-modified pastes are most plausibly attributed to the high reactivity of potassium-rich phases, particularly those previously identified in the mineralogical analysis. To the best of our knowledge, this is the first report to document such behavior in potassium-rich biomass fly ash systems used in 3D-printable cementitious formulations.

The calorimetry curves for APA-modified systems (Figure 10b,e) exhibit an increase in the intensity of the main hydration peak compared to WPC, which presents a conventional hydration profile with a moderate peak centered around 6 h. In contrast, mixtures A1 through A4 display significantly higher peak intensities with slightly earlier peak times. APA functions as a hydration accelerator by enhancing clinker-phase dissolution and promoting the early formation of calcium silicate hydrate (C–S–H). The incorporation of air-entraining and dispersing agents significantly increases early ionic mobility, shortens the induction period, and intensifies the overall hydration rate [39].

Cumulative heat release data further confirms the accelerating effect of APA. All APA-containing mixtures exhibit greater total heat release than WPC, with the A4 mixture reaching over 320 J/g at 30 h. This increase in cumulative heat is indicative of a higher degree of hydration, driven by enhanced ion mobility and nucleation efficiency facilitated by APA. The steep slope observed during the first 30 min (inset of Figure 10b) underscores APA’s role in promoting the rapid dispersion and early dissolution of reactive phases.

When APA is combined with BFAK in ternary mixtures (OPT-series), the magnitude of the main hydration peak increases substantially compared to the WPC reference (Figure 10c,f), reinforcing the synergistic stimulation of the hydration process. This enhancement aligns with prior findings: APA improves ion transport and nucleation, while BFAK contributes potassium ions that promote early C–S–H development. However, a deviation is noted in the temporal evolution of the heat release. Despite the heightened peak intensities, the main hydration peaks in OPT-series pastes appear later than in WPC. This shift indicates a prolonged acceleration induction period. The extension of the induction period suggests that the high K_2_O content from BFAK may counteract APA’s accelerating action by temporarily stabilizing a passivating layer or altering pore solution saturation. These effects delay the breakdown of protective films around clinker grains, thus postponing the onset of rapid hydration, which is consistent with the delayed setting times observed in Table 4.

Table 4 lists the formulations that show normal consistency for 3D printing and the corresponding setting times, determined by the Vicat apparatus. All mixtures containing APA and BFAK exhibit longer setting times than the WPC reference, thereby confirming the trends observed in the calorimetric analysis. A comparative analysis of APA dosage (e.g., OPT/OPT2-1% vs. OPT1/OPT3-1.5%) reveals that a higher APA content tends to reduce the setting time. This is also due to the plasticizing action of APA, which allows a normal consistency to be achieved while using less water. Conversely, BFAK content (e.g., OPT/OPT1-3% vs. OPT2/OPT3-2%) appears to slightly extend the induction period, consistent with previous findings for BFAK-containing cement pastes (B2–B3).

The interval between the initial and final setting times—commonly referred to as the “open working window”—serves as a practical indicator of the timeframe available for shaping and forming printed elements. The WPC formulation exhibits the narrowest window at approximately 90 min. In contrast, APA- and BFAK-modified formulations such as OPT, OPT1, and OPT2 demonstrate considerably extended working times in the range of 160–180 min. The OPT3 mixture displays an intermediate open window of approximately 148 min.

### 3.3. Evaluation of Workability and Structural Performance of Mortar for 3D Printing

**Figure 11:**
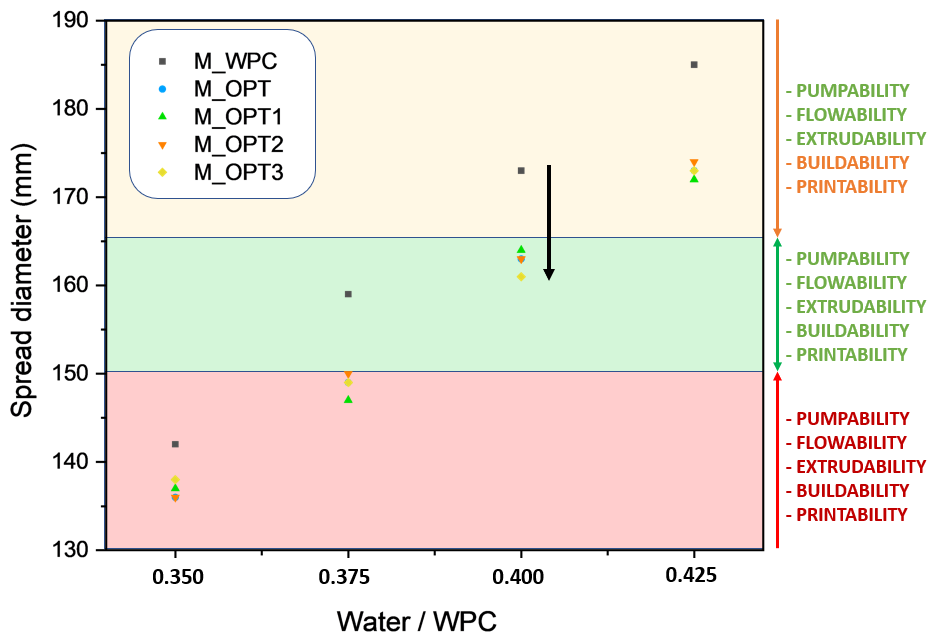
Mini-slump diameter of mortar mixtures at different water-to-WPC cement ratios and workability zone.

Workability was assessed through the mini-slump spread diameter test, a reliable and widely used method for evaluating the fresh-state rheological behavior of cementitious mixtures. Figure 11 presents the spread diameters of all tested formulations plotted against the water-to-WPC (white Portland cement) ratio, expressed in g H_2_O/g WPC. Across all water/WPC ratios, the optimized formulations consistently exhibited lower spread values than the reference mortar, reflecting the influence of rheology-modifying additives.

This behavior is attributed to the stiffening effect introduced by the addition of BFAK and APA, as corroborated by rheological measurements. These additives increase yield stress and thixotropy, which improve shape retention and reduce deformation. This observation is in agreement with Suvash Chandra [40], who demonstrated that higher yield stress enhances print stability by minimizing layer collapse in 3D-printed mortars. As detailed by Lixiong Cai [41], the mini-slump test enables quantitative identification of fresh-state conditions suitable for extrusion-based printing. Notably, Yi Wei [42] identified critical thresholds for printability: mini-slump values below 145–150 mm correspond to unprintable mixtures due to excessive stiffness, often caused by low W/B and high S/B ratios. Conversely, values near 180 mm are associated with over-fluidity, leading to insufficient structural build-up.

Within this framework, the optimal mini-slump range was fixed for 3D-printable mortars as between 150 and 170 mm. Mortars within this range achieve a critical balance between extrudability and buildability: the mixtures are sufficiently fluid to allow continuous flow through the nozzle yet cohesive enough to retain their form once deposited. Based on these findings, a water/WPC ratio of 0.40 was selected for the calibration cylinder tests, as it reliably produced mixtures within the optimal mini-slump window. It may be important to emphasize the relevance of controlling the preparation of the formulations, given that variations as small as 0.025, i.e., 2.5% in the water content, result in significant changes in the consistency of the mixtures. This zone, referred to as the “green zone,” satisfies the essential criteria for successful 3D printing: pumpability, flowability, extrudability, buildability, and printability. By contrast, the “orange zone” encompasses suboptimal conditions, in which deposition is possible but layer stacking is limited due to poor structural integrity. The “red zone” represents insufficient workability, where material flow is constrained, rendering the mixture unprintable, as shown in Figure 11. At a water/WPC ratio of 0.40, all optimized mixtures demonstrated stable extrusion and acceptable buildability.

To assess structural performance, calibration cylinders were printed vertically to determine maximum buildable height under static load. As shown in Figure 11, the reference mortar (M-WPC) failed to retain shape at the selected water ratio, undergoing significant deformation. This result reinforces the need for tailored formulations in 3D-printing applications.

**Figure 12:**
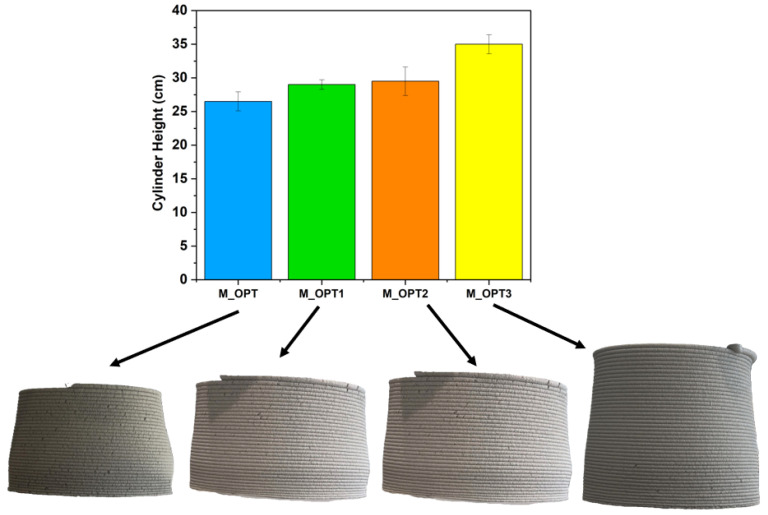
Correlation between cylinder height and structural collapse of 3D-printed mortars prepared with water/WPC = 0.4.

Dual-modified mortars containing both APA and BFAK demonstrated marked improvements in buildability. Among these, formulations with higher APA content, specifically M-OPT1 and M-OPT3, each incorporating 1.5% APA, achieved notably greater cylinder heights than M-OPT and M-OPT2, which contained 1.0% APA, as shown in Figure 12. The performance of the printed calibration cylinders was closely linked to the rheological parameters previously measured in Section 3.1.1. Formulations exhibiting intermediate yield stress and enhanced thixotropic area demonstrated superior buildability and structural retention, maintaining verticality and dimensional consistency throughout the printing process. These rheological characteristics ensured a stable layer-by-layer deposition without collapse, correlating directly with the successful outcomes observed in Figure 12. Additionally, the results indicate that mortars incorporating 2% BFAK consistently achieved greater build heights than those formulated with 3% BFAK. Therefore, while BFAK enhances rheological and hydration behavior at moderate levels, excessive addition can counteract these benefits by compromising structural cohesion and stacking stability. These findings validate the role of rheological parameters in predicting printability and shape retention, reinforcing the importance of thixotropy and yield stress optimization during mixture design.

M-OPT3 was the best-performing mixture, achieving the greatest height with minimal deformation and highly cohesive layering. This aligns with rheological data in Section 3.1.1 and Section 3.1.2, where M-OPT3 exhibited the highest yield stress and thixotropic behavior among the tested samples. The strong agreement between rheological performance and structural buildability supports the use of rheological pre-screening as a predictive tool in the formulation of 3D-printable mortars.

In addition to the structural buildability assessment, mechanical tests performed on the optimized mixtures confirmed that neither compressive nor flexural strength was significantly altered by the inclusion of APA and BFAK. The measured strength values remained comparable to those obtained for the reference mortar without any additives or ashes, indicating that the incorporation of these components does not compromise the mechanical integrity of the printed material.

## 4. Conclusions

This study explored the innovative use of potassium-rich biomass fly ash (BFAK) as an additive in cementitious mortars for 3D printing. The results demonstrated the potential of BFAK to control the rheological properties and structural performance of the mixtures. The yield stress and thixotropy were increased with the addition of BFAK, confirming its role in improving structural stability after extrusion. Higher BFAK content leads to enhanced material cohesion, reducing deformation risks under self-weight and ensuring better retention of the printed shape. The synergy between BFAK and APA further improves the material’s ability to maintain layer integrity, enabling better layer-by-layer stacking without excessive deformation. BFAK-modified mortars exhibit a reduction in spread diameter in flow table tests, ensuring lower material dispersion upon deposition. An addition of 2–3.0 wt.% BFAK ensures a correct balance between extrudability and buildability, where the material retains adequate deformation resistance while remaining pumpable and extrudable. Samples show adequate deformation resistance while remaining pumpable. The combined effect of BFAK with APA enhances printability, with APA counteracting the excessive viscosity gain given by BFAK. The printing of large-scale calibration cylinders validated the impact of BFAK on material buildability. The highest printable height (35 cm) was achieved with M_OPT3 (1.5% APA, 2% BFAK), demonstrating better weight-bearing capacity. The findings of the present investigation might ensure waste valorization, while contributing towards the deployment of innovative manufacturing techniques.

## Figures and Tables

**Table 1:** Oxide contents for raw materials WPC and BFAK, determined by XRF.

Raw Material	Oxide Content (%wt)												
	SiO_2_	Al_2_O_3_	Fe_2_O_3_	CaO	MgO	SO_3_	Na_2_O	K_2_O	TiO_2_	P_2_O_5_	Cl	LOI	Total
WPC	22.5	5.2	0.3	66.5	0.9	2.5	0.1	0.1	–	–	0.1	1.5	99.7
BFAK	15.7	2.9	1.5%	6.8	3.4	5.1	0.7	45.2	0.2	3.7	4.4	9.6	99.2

**Table 2:** Formulations of cementitious pastes tested (w/c = 0.4).

Mixture	WPC (g)	Additives (g/100 g WPC)	
		APA	BFAK
WPC	100.0	0.0	0.0
A1	100.0	0.5	0.0
A2	100.0	1.0	0.0
A3	100.0	1.5	0.0
A4	100.0	2.0	0.0
B1	100.0	0.0	1.0
B2	100.0	0.0	2.0
B3	100.0	0.0	3.0
B4	100.0	0.0	4.0
OPT	100.0	1.0	3.0
OPT1	100.0	1.5	3.0
OPT2	100.0	1.0	2.0
OPT3	100.0	1.5	2.0

**Table 3:** Formulations of mortar mixtures tested in 3D printing.

Mixture	WPC (%wt)	LS (%wt)	Additives (g/100 g WPC)	
			APA	BFAK
M_WPC	62.5	37.5	0.0	0.0
M_OPT	62.5	37.5	1.0	3.0
M_OPT1	62.5	37.5	1.5	3.0
M_OPT2	62.5	37.5	1.0	2.0
M_OPT3	62.5	37.5	1.5	2.0

**Table 4:** w/c ratio required to achieve normal consistency of selected formulations’ corresponding setting times.

Mixture	Normal Consistency w/c (%wt)	Setting Time (min)	
		Initial	Final
WPC_NC	30.5	87.0	175.0
OPT_NC	29.5	125.0	305.0
OPT1_NC	28.5	116.0	277.0
OPT2_NC	29.5	164.0	319.0
OPT3 _NC	28.8	156.0	304.0

## Data Availability

The original contributions presented in this study are included in the article. Further inquiries can be directed to the corresponding author.
